# Performance of ChatGPT-4 on Taiwanese Traditional Chinese Medicine Licensing Examinations: Cross-Sectional Study

**DOI:** 10.2196/58897

**Published:** 2025-03-19

**Authors:** Liang-Wei Tseng, Yi-Chin Lu, Liang-Chi Tseng, Yu-Chun Chen, Hsing-Yu Chen

**Affiliations:** 1 Division of Chinese Acupuncture and Traumatology, Center of Traditional Chinese Medicine, Chang Gung Memorial Hospital, Taoyuan, Taiwan; 2 Division of Chinese Internal Medicine, Center for Traditional Chinese Medicine, Chang Gung Memorial Hospital, No. 123, Dinghu Rd, Gueishan Dist, Taoyuan, 33378, Taiwan, 886 3 3196200 ext 2611, 886 3 3298995; 3 Google International LLC Taiwan Branch, Taipei, Taiwan; 4 School of Medicine, Faculty of Medicine, National Yang-Ming Chiao Tung University, Taipei, Taiwan; 5 Taipei Veterans General Hospital, Yuli Branch, Taipei, Taiwan; 6 Institute of Hospital and Health Care Administration, National Yang-Ming Chiao Tung University, Taipei, Taiwan; 7 School of Traditional Chinese Medicine, College of Medicine, Chang Gung University, Taoyuan, Taiwan

**Keywords:** artificial intelligence, AI language understanding tools, ChatGPT, natural language processing, machine learning, Chinese medicine license exam, Chinese medical licensing examination, medical education, traditional Chinese medicine, large language model

## Abstract

**Background:**

The integration of artificial intelligence (AI), notably ChatGPT, into medical education, has shown promising results in various medical fields. Nevertheless, its efficacy in traditional Chinese medicine (TCM) examinations remains understudied.

**Objective:**

This study aims to (1) assess the performance of ChatGPT on the TCM licensing examination in Taiwan and (2) evaluate the model’s explainability in answering TCM-related questions to determine its suitability as a TCM learning tool.

**Methods:**

We used the GPT-4 model to respond to 480 questions from the 2022 TCM licensing examination. This study compared the performance of the model against that of licensed TCM doctors using 2 approaches, namely direct answer selection and provision of explanations before answer selection. The accuracy and consistency of AI-generated responses were analyzed. Moreover, a breakdown of question characteristics was performed based on the cognitive level, depth of knowledge, types of questions, vignette style, and polarity of questions.

**Results:**

ChatGPT achieved an overall accuracy of 43.9%, which was lower than that of 2 human participants (70% and 78.4%). The analysis did not reveal a significant correlation between the accuracy of the model and the characteristics of the questions. An in-depth examination indicated that errors predominantly resulted from a misunderstanding of TCM concepts (55.3%), emphasizing the limitations of the model with regard to its TCM knowledge base and reasoning capability.

**Conclusions:**

Although ChatGPT shows promise as an educational tool, its current performance on TCM licensing examinations is lacking. This highlights the need for enhancing AI models with specialized TCM training and suggests a cautious approach to utilizing AI for TCM education. Future research should focus on model improvement and the development of tailored educational applications to support TCM learning.

## Introduction

Traditional Chinese medicine (TCM), recognizeartid as one of the most renowned traditional medical systems, boasts a history spanning thousands of years. In the modern era, TCM has evolved to form an integral part of the formal health care system in East Asian countries, particularly in China and Taiwan [1,2]. TCM encompasses a wealth of theoretical knowledge and features unique diagnostic and treatment methods, such as acupuncture and herbal therapy. As a highly practical discipline, TCM learning traditionally relies on the accumulation of experience and the mentorship inherent in the master-apprentice system; hence, this education model may not be sufficiently reliable or comprehensive. However, with the emerging need for integrative medicine over time, TCM has been integrated into the modern medical education system. This integration has led to prominent changes in educational approaches. The incorporation of TCM into academic institutions resulted in the establishment of formal examination systems. For instance, in Taiwan, TCM practitioners must pass a biannual licensing examination, termed the National Senior Professional and Technical Examinations for Chinese Medicine Practitioners (hereinafter called the “TCM licensing examinations”), to practice as a licensed TCM doctor, similar to their Western medicine counterparts [3].

The advancements in technology and the development of artificial intelligence (AI) have begun to impact and challenge the medical field, with TCM being no exception [4,5]. In the past year, significant progress has been made in AI language models, particularly those based on the generative pretrained transformer (GPT) architecture. ChatGPT, a conversational variant of the GPT model, has demonstrated its potential across various domains [6]. Recognized for its foundational medical knowledge and conversational capabilities, ChatGPT is considered a valuable tool in medical education, aiding in the understanding and application of medical knowledge [7], thereby facilitating student learning [8]. However, its responses are not consistently reliable. Unlike humans who answer questions based on an understanding of the content, it generates replies by drawing from a vast database. Therefore, although it can produce human-like conversations and respond to inquiries, it cannot guarantee the accuracy of its responses [9,10].

Discussions have emerged regarding the sufficiency of AI for clinical decision-making and basic medical consultation [7,11]. In addition, to be a potential mentor for medical students, one benchmark is the ability of AI to pass national licensing examinations (the minimum standard for practicing physicians). Thus, the application of ChatGPT in medical examinations has opened a new research direction. Studies have shown that GPT models, especially GPT-4, can achieve commendable scores on a variety of standardized tests for multiple professions, such as physicians [12-14], pharmacists [15], and nurses [16]. This success in examination settings has sparked interest in the potential of ChatGPT as a self-learning tool, suggesting its use for examination preparation and knowledge enhancement [17].

As previously mentioned, while TCM is a traditional medical system distinct from modern medicine, it has been integrated into modern medical education systems and subjected to formal examinations. The question arises: does ChatGPT possess the requisite knowledge level to assist TCM students in their learning? Only 1 study examined GPT’s ability to answer TCM questions, but it focused on questions sourced from online TCM texts rather than formally recognized examination questions and utilized older GPT models (GPT-3 Turbo) [18]. In contrast, a more rigorous study on traditional Korean medicine found that, due to the unique nature of traditional medicine, GPT models require specially optimized prompts, such as language-related adjustments, to pass examinations [19]. However, considering the classical Chinese language barrier and different medical theories in TCM, whether GPT models would face challenges in TCM licensing examinations remains unexplored.

The aim of this study is to evaluate whether ChatGPT can accurately understand and respond to TCM questions by assessing its performance in simulated examination environments. By analyzing the accuracy of AI-generated answers, we sought to identify factors affecting their correctness. This study also aims to understand the consistency between AI-generated answers and their accompanying explanations, offering insights into the depth of understanding of this model. By analyzing the performance of ChatGPT in simulated TCM licensing examinations and comparing it with human performance, this study hopes to provide new insights and recommendations for innovation and development in TCM education.

## Methods

### Study Design

**Figure 1.**
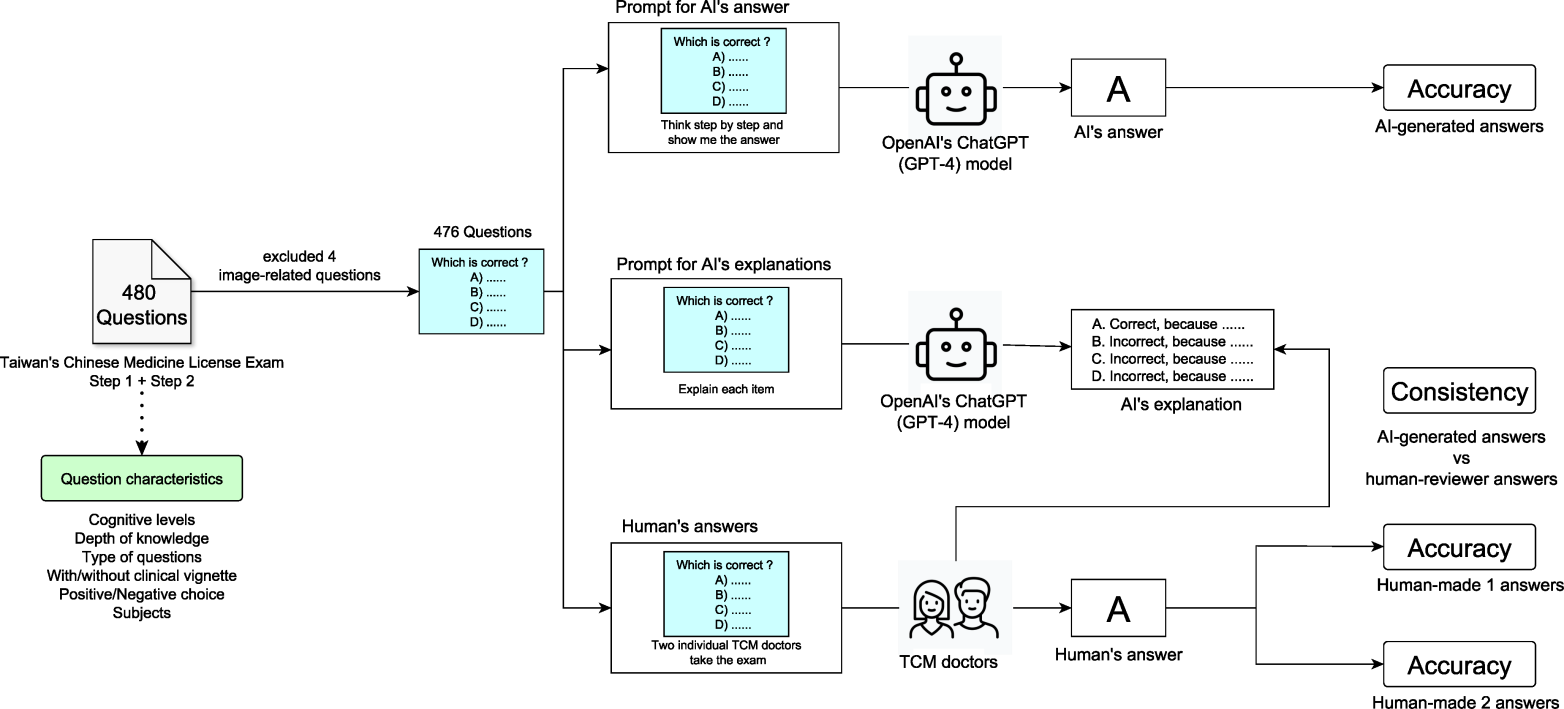
Flowchart of this study. GPT: generative pretrained transformer; TCM: traditional Chinese medicine.

Figure 1 shows the data processing flowchart of this study. The feasibility of using ChatGPT (GPT-4 model, with a knowledge cutoff date of September 2021), developed by OpenAI, with 2 different prompts on responding to the first National Senior Professional and Technical Examinations for Chinese Medicine Practitioners was assessed by comparing the responses of the model to those of licensed TCM resident doctors. A total of 480 questions from the 2022 examination were inputted into ChatGPT, and 2 different approaches were used to obtain responses from ChatGPT. The first step involved prompting AI to select the correct answer directly from the question options. The second step required ChatGPT to explain why each option was correct or incorrect before selecting the correct answer. For the second step, individual answers and explanations from ChatGPT were manually assessed for accuracy and consistency. Subsequently, accuracy was measured by comparing the AI-selected answers with the correct answers. Additionally, the performance of AI was benchmarked against that of human experts. Two individual TCM resident doctors took the same examination without preparation, and their answers were also evaluated for accuracy. Finally, consistency was evaluated by comparing explanations against a standard set of answers for logical coherence, and the reasons for inconsistency were also verified by the 2 TCM doctors.

### The TCM Licensing Examination in Taiwan

In Taiwan, TCM doctors are qualified through 2 stages of licensing examinations after graduation from their TCM course at the university. The contents and answers are freely downloadable after each examination from the following website [20]. The examinations contain 2 stages corresponding to 10 subjects. The first stage consists of basic theory, including 黃帝內經 (Huangdi Neijing), 難經 (Nanjing) (domain I), and basic pharmacology and formulation (domain II). The second stage consists of principles of diagnosis and treatment, including 傷寒論 (Shanghanlun) and 金匱要略 (Jinguiyaolue) (domain III), TCM internal medicine (domain IV), TCM gynecology and obstetrics (domain IV), TCM pediatrics (domain IV), TCM dermatology (domain V), TCM otorhinolaryngology (domain V, including questions regarding the specialty concerning ears, nose, and throat [ENT]) and ophthalmology (domain V), TCM traumatology (domain V), and acupuncture (domain VI). Each domain contains 80 multiple-choice questions with single answers. The full score of each domain is 100. The examination score is calculated by dividing the total score by the number of subjects. Only examinees obtaining average scores ≥60 pass the examination. TCM students are eligible to take the first-stage examination when they have earned the requisite fourth-year university credits. Before the second-stage examination, TCM students must first pass the first-stage examination and graduate from the 7-year university course.

### Question Characteristics

A total of 5 factors were used to characterize the examination questions, including the cognitive level, depth of knowledge (DOK), type of questions, vignette style, and polarity of questions (Table S1 in Multimedia Appendix 1). LWT and YCL independently reviewed and classified all questions according to the definitions of these 5 factors. In case of disagreement, HYC was consulted, and the disagreement was resolved by reaching a consensus among all authors. Bloom’s taxonomy was modified to classify the questions into lower-order thinking skills (LOTS) and higher-order thinking skills (HOTS). LOTS include remembering, understanding, and applying knowledge to questions, while HOTS include further analyzing, evaluating, and creating after learning [21,22]. For the DOK, 3 levels, ranging from low to high based on Webb’s framework on science, were defined as recall, concept, and strategic thinking. Questions with higher levels of DOK indicate the recruitment of sophisticated thinking [23]. Furthermore, the licensing examinations in Taiwan are presented as single-choice questions, adhering to the 1 stem, 4 choices policy. However, 2 types of questions were used to add variety to examination questions, including single-answer multiple-choice (SAMC) and single-answer, multiple-response multiple-choice (SAMRMC) questions. SAMC questions had only 1 most appropriate answer, while SAMRMC questions require the tester to choose the most appropriate answer composed of multiple correct options provided in each question (Table S2 in Multimedia Appendix 2). Moreover, if the content of a question presents clinical scenarios, this question would be categorized as the clinical vignette type. This type of question typically aims to examine the ability of the tester to analyze the clinical conditions and corresponding actions. The polarity of a question depended on whether the question was positively or negatively framed. A “positive-choice question” solicits the correct or affirmative answer, whereas a “negative choice question” demands the identification of the incorrect or negative answer.

### Prompt for AI-Generated Answers

To enhance the precision and brevity of responses obtained from ChatGPT (GPT-4 model), we strategically added “think step-by-step” to our queries. This approach aimed to guide the model toward a methodical and sequential problem-solving process. Subsequently, by integrating the command “but show me only the answer, do not explain it,” we aimed to extract a more refined and consolidated answer, significantly boosting the response accuracy of the model. An example of a prompt with response is demonstrated in Table S3 in Multimedia Appendix 3. We created a collection of unique prompts derived from an equal number of questions in the question database, submitting them sequentially to the AI model. To solve the issue of memory retention between submissions, we used a specialized application designed to initiate separate application programming interface requests for each prompt. This approach guaranteed that each application programming interface interaction would be initiated separately. This ensures that the processing of each prompt and the generation of its answer were conducted in isolation, thereby preserving the integrity of the responses without interference from a prior response [24,25].

### Prompt for Explanations Provided By AI Through Step-By-Step and Human-Curated Answers

Furthermore, to understand the thinking process of GPT and evaluate the accuracy of its interpretation of our inquiries, we prompted ChatGPT to “explain each item” for each question. This prompt directed the AI to furnish exhaustive explanations for each item [26] (Table S4 in Multimedia Appendix 4). LWT and YCL reviewed all explanations to items and reached decisive responses based on AI-generated explanations. This process was termed “human-curated responses.” To authentically represent the logic of AI, we refrained from making any human amendments, even if the explanations provided by AI were incorrect. The answer would be marked as “wrong” if the AI-generated explanations were incorrect.

### Outcome Assessment

We evaluated the accuracy of answers generated by the GPT, those made by humans, and explanations provided by the GPT and curated by humans. This was achieved by calculating the ratio of accurate responses to the total number of questions and representing the results as a percentage. This measure of accuracy underwent comparative analysis across different attributes of the questions. The human-curated answers, which encapsulated the interpretation of questions by AI, were evaluated by LWT, YCL, and HYC, who reached a consensus to identify instances of misinterpretation of the question (GPT cannot understand the question and does not provide an answer), misunderstanding of concepts (GPT can understand the question, but lacks knowledge of the topic), and incorrect application of principles (the responses GPT provides are correct in general but fail to answer the question).

### Statistical Analysis

Proportions and percentages were used to present categorical data. A logistic regression approach was adopted to assess the effect of various attributes of questions on the correctness of responses generated by GPT-4. The cognitive complexity of the questions, their structural format, the inclusion of clinical vignettes, the overall polarity of questions, and the subjects were used as covariates in the logistic regression with univariable and multivariable models. The influence of each variable on the probability of the AI producing accurate answers was quantified using the adjusted odds ratio, accompanied by 95% CIs. Additionally, the κ statistic was used to evaluate the agreement between responses generated by GPT and curated by humans. This represented the different viewpoints concerning the same explanation between GPT and humans. *P*<.05 was used as the threshold for statistical significance. All statistical evaluation was performed utilizing Stata 17 (StataCorp LLC).

### Ethical Considerations

This study did not require ethical approval, as it analyzed data obtained from a publicly available database. The test questions and answers used were originally created and copyrighted by the Taiwan Ministry of Examination and made accessible for academic research purposes. The Ministry retains full copyright over the examination content and confirmed that this research adhered to copyright regulations without any infringement.

## Results

### Question Characteristics

**Figure 2.**
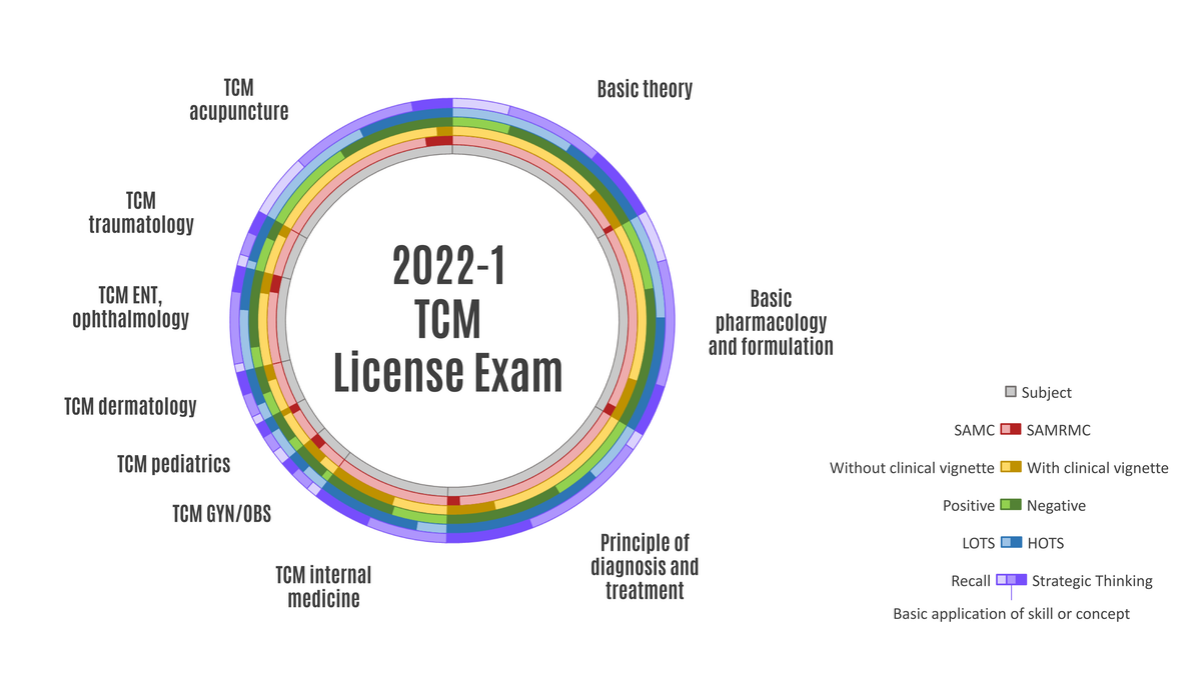
Distribution of subjects in TCM licensing examinations. The detailed numbers and proportion of each subject’s question types can be seen in Table 1. ENT: ears, nose, and throat; GYN/OBS: gynecology/obstetrics; HOTS: higher-order thinking skills; LOTS: lower-order thinking skills; SAMC: single-answer multiple-choice; SAMRMC: single-answer, multiple-response multiple-choice; TCM: traditional Chinese medicine.

The examination encompassed a total of 480 questions spanning 10 specialties. Four image-related questions were excluded. Our findings indicated that most questions were HOTS, SAMC, negative-choice, and without a clinical vignette. According to Bloom’s taxonomy of cognitive learning, the majority of questions across all subjects required HOTS (263/476, 55.3%; LOTS: 213/476, 44.7%). In particular, principles of diagnosis and treatment, TCM internal medicine, TCM dermatology, and TCM traumatology predominantly featured HOTS (58/80, 72.5%; 37/48, 77.1%; 13/19, 68.4%; and 17/20, 85%, respectively), while TCM pediatrics mainly involved LOTS (11/16, 68.8%). Within the LOTS category, “remembering” was the most common type (121/213, 56.8%), while “analyzing” dominated the HOTS category (255/263, 97%). In terms of Webb’s DOK analysis of question types, the basic application of skill/concept represented the largest proportion (248/476, 52.1%), surpassing recall (85/476, 17.9%) and strategic thinking (143/476, 30%). A large portion of the questions were formatted as SAMC (439/476, 92.2%). Negative-choice questions comprised 62.2% (296/476) of the total, while 23.9% (180/476) of the questions included a clinical vignette (Table 1, Figures2  3).

**Table 1.** Characteristics of TCM^a^ licensing examinations in Taiwan, 2022.

Cognitive level	Total (n=476)	Basic theory(n=80)	Basic pharmacology and formulation (n=80)	Principle of diagnosis and treatment (n=80)	TCM internal medicine (n=48)	TCM GYN/OBS^b^ (n=16)	TCM pediatrics (n=16)	TCM dermatology (n=19)	TCM ENT, ophthalmology (n=37)	TCM traumatology (n=20)	TCM acupuncture (n=80)
**LOTS** ^**c**^											
Remembering	121 (25.4)	28 (35)	19 (23.8)	7 (8.8)	1 (2.1)	6 (37.5)	8 (50)	5 (26.3)	13 (35.1)	3 (15)	31 (38.8)
Understanding	41 (8.6)	10 (12.5)	10 (12.5)	7 (8.8)	2 (4.2)	0 (0)	0 (0)	0 (0)	5 (13.5)	0 (0)	7 (8.8)
Applying	51 (10.7)	7 (8.8)	9 (11.3)	8 (10)	8 (16.7)	3 (18.8)	3 (18.8)	1 (5.3)	4 (10.8)	0 (0)	8 (10)
**HOTS** ^**d**^											
Analyzing	255 (53.6)	34 (42.5)	40 (50)	54 (67.5)	37 (77.1)	7 (43.8)	5 (31.3)	13 (68.4)	15 (40.5)	17 (85)	33 (41.3)
Evaluating	8 (1.7)	1 (1.3)	2 (2.5)	4 (5.0)	0 (0)	0 (0)	0 (0)	0 (0)	0 (0)	0 (0)	1 (1.3)
**Depth of knowledge**											
Recall	85 (17.9)	20 (25)	18 (22.5)	6 (7.5)	0 (0)	4 (25)	5 (31.3)	3 (15.8)	3 (8.1)	4 (20)	22 (27.5)
Basic application of skill/concept	248 (52.1)	34 (42.5)	44 (55)	44 (55)	28 (58.3)	7 (43.8)	6 (37.5)	8 (42.1)	25 (67.6)	8 (40)	44 (55)
Strategic thinking	143 (30)	26 (32.5)	18 (22.5)	30 (37.5)	20 (41.7)	5 (31.3)	5 (31.3)	8 (42.1)	9 (24.3)	8 (40)	14 (17.5)
**Type of question options and choices**											
SAMC^e^	439 (92.2)	78 (97.5)	76 (95)	75 (93.8)	48 (100)	11 (68.8)	13 (81.3)	19 (100)	30 (81.1)	20 (100)	69 (86.3)
SAMRMC^f^	37 (7.8)	2 (2.5)	4 (5)	5 (6.3)	0 (0)	5 (31.3)	3 (18.8)	0 (0)	7 (18.9)	0 (0)	11 (13.8)
**Clinical vignette**											
Without clinical vignette	362 (76.1)	63 (78.8)	63 (78.8)	61 (76.3)	22 (45.8)	7 (43.8)	14 (87.5)	13 (68.4)	29 (78.4)	16 (80)	74 (92.5)
With clinical vignette	114 (23.9)	17 (21.3)	17 (21.3)	19 (23.8)	26 (54.2)	9 (56.3)	2 (12.5)	6 (31.6)	8 (21.6)	4 (20)	6 (7.5)
**Polarity of question options**											
Positive	180 (37.8)	22 (27.5)	27 (33.8)	36 (45)	21 (43.8)	3 (18.8)	5 (31.3)	9 (47.4)	8 (21.6)	13 (65)	36 (45)
Negative	296 (62.2)	58 (72.5)	53 (66.3)	44 (55)	27 (56.3)	13 (81.3)	11 (68.8)	10 (52.6)	29 (78.4)	7 (35)	44 (55)

a TCM: traditional Chinese medicine.

b GYN/OBS: gynecology/obstetrics.

c LOTS: lower-order thinking skills.

d HOTS: higher-order thinking skills.

e SAMC: single-answer multiple-choice.

f SAMRMC: single-answer, multiple-response multiple-choice.

**Figure 3.**
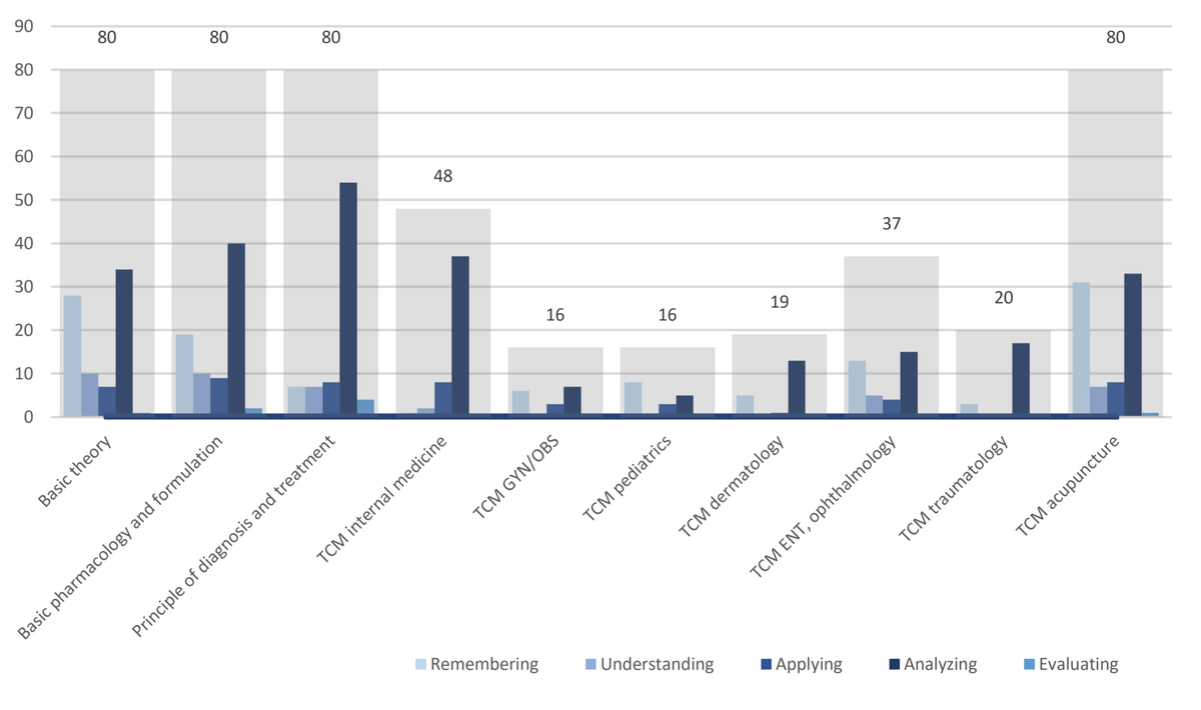
Analysis of question types according to Bloom’s cognitive level in TCM licensing examinations. ENT: ears, nose, and throat; TCM: traditional Chinese medicine.

### GPT-4 Model Performance and Accuracy Across Different Question Characteristics

**Figure 4.**
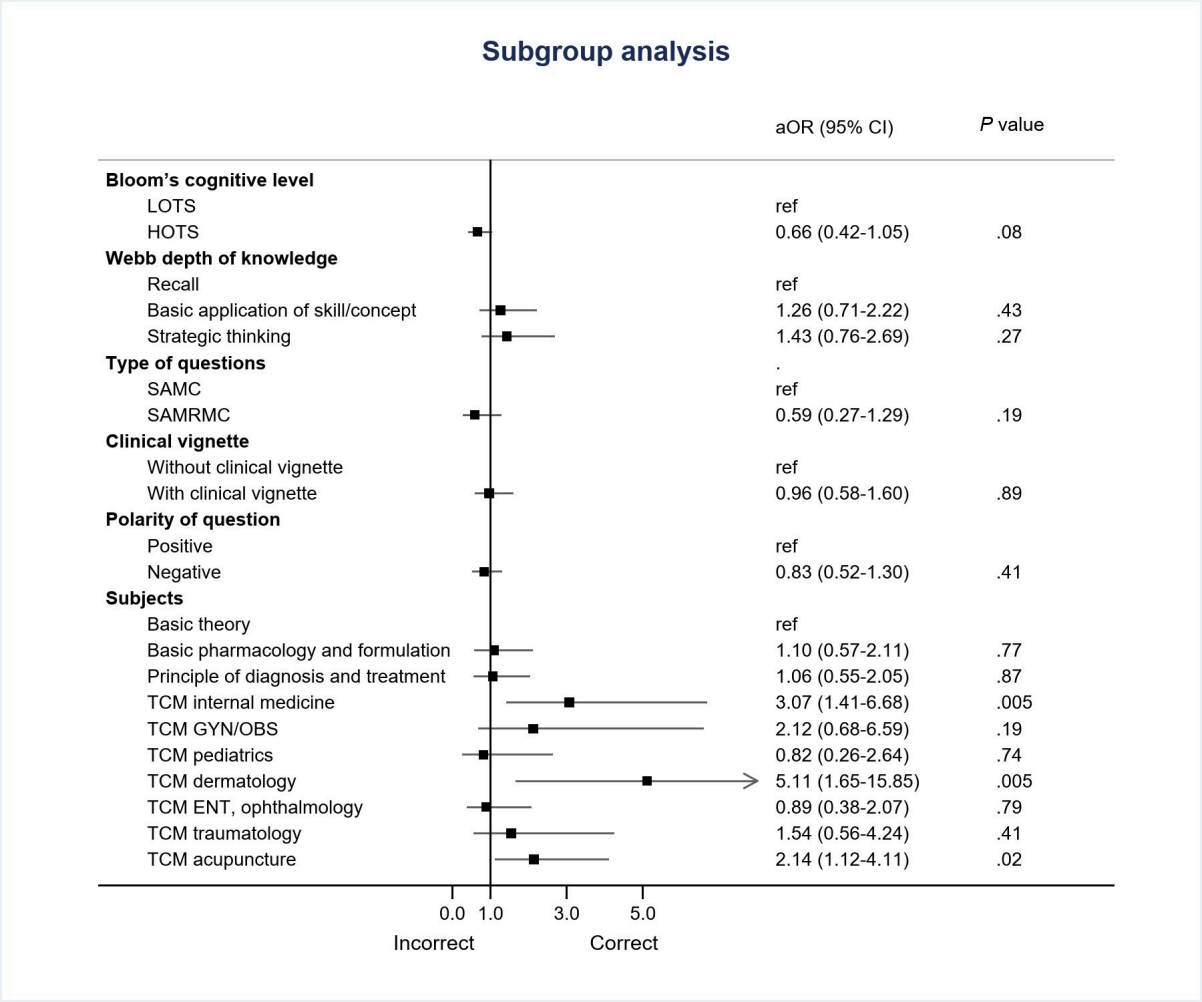
Factors associated with correct answers provided by ChatGPT-4. aOR: adjusted odds ratio; ENT: ears, nose, and throat; GYN/OBS: gynecology/obstetrics; HOTS: higher-order thinking skills; LOTS: lower-order thinking skills; SAMC: single-answer multiple-choice; SAMRMC: single-answer, multiple-response multiple-choice; TCM: traditional Chinese medicine.

We observed that the performance of the GPT-4 model was inferior to that of humans and did not demonstrate significant variation across different categories of examination questions. The GPT-4 model demonstrated an overall accuracy of only 43.9% (209/476). In comparison, 2 human evaluators achieved accuracy rates of 70% (333/476) and 78.4% (373/476), respectively (Table 2). The performance of ChatGPT across various variables is shown in Table 3. The accuracy of AI-generated answers did not show a significant correlation with the characteristics of the questions, regardless of the classification method used (Figure 4). The GPT-4 model demonstrated a performance close to that of humans in TCM dermatology and TCM traumatology. The accuracy of AI-generated answers varied among the test subjects, ranging from 31.3% in TCM pediatrics to 73.7% in TCM dermatology. Notably, only TCM internal medicine (adjusted odds ratio [aOR] 3.07, 95% CI 1.41‐6.68; *P*=.005), TCM dermatology (aOR 5.11, 95% CI 1.65‐15.85; *P*=.005), and TCM acupuncture (aOR 2.14, 95% CI 1.12‐4.11; *P*=.02) showed statistically significant better performance (Figure 4). On the other hand, GPT had a higher, but not statistically significant, accuracy rate for questions categorized as LOTS (96/213, 45.1%), SAMC (197/439, 44.9%), strategic thinking (66/143, 46.2%), with clinical vignette (52/114, 45.6%), and positive-choice (85/180, 47.2%).

**Table 2.** Accuracy rates of testers and ChatGPT-4 for TCM^a^ licensing examinations.

	Number of questions	Number of correct responses	Accuracy, %
Human-made 1	476	333	70
Human-made 2	476	373	78.4
ChatGPT-4^b^	476	209	43.9
Human-curated answer 1	476	192	40.3
Human-curated answer 2	476	186	39.1

a TCM: traditional Chinese medicine.

b ChatGPT did not show answers to 7 questions although an explanation was provided.

**Table 3.** Accuracy rates of testers and ChatGPT-4 across different types and subjects of questions.

	Accuracy, %				
	Human-made 1	Human-made 2	ChatGPT-4	Human-curated 1	Human-curated 2
**Bloom’s cognitive level**					
LOTS^a^	150 (70.4)	164 (77)	96 (45.1)	78 (36.6)	75 (35.2)
HOTS^b^	183 (69.6)	209 (79.5)	113 (43)	114 (43.3)	111 (42.2)
**Depth of knowledge**					
Recall	57 (67.1)	65 (76.5)	34 (40)	27 (31.8)	22 (25.9)
Basic application of skill/concept	172 (69.4)	193 (77.8)	109 (44)	103 (41.5)	102 (41.1)
Strategic thinking	104 (72.7)	115 (80.4)	66 (46.2)	62 (43.4)	62 (43.4)
**Type of questions**					
SAMC^c^	312 (71.1)	346 (78.8)	197 (44.9)	180 (41)	176 (40.1)
SAMRMC^d^	21 (56.8)	27 (73)	12 (32.4)	12 (32.4)	10 (27)
**Vignette style question**					
Without clinical vignette	248 (68.5)	283 (78.2)	157 (43.4)	143 (39.5)	137 (37.8)
With clinical vignette	85 (74.6)	90 (78.9)	52 (45.6)	49 (43)	49 (43)
**Polarity of question**					
Positive	129 (71.7)	142 (78.9)	85 (47.2)	78 (43.3)	76 (42.2)
Negative	204 (68.9)	231 (78)	124 (41.9)	114 (38.5)	110 (37.2)
**Subjects**					
Basic theory	51 (63.7)	63 (78.8)	29 (36.3)	29 (36.3)	28 (35)
Basic pharmacology and formulation	63 (78.8)	66 (82.5)	30 (37.5)	32 (40)	28 (35)
Principle of diagnosis and treatment	57 (71.3)	58 (72.5)	29 (36.3)	29 (36.3)	29 (36.3)
TCM^e^ internal medicine	41 (85.4)	44 (91.7)	30 (62.5)	24 (50)	24 (50)
TCM gynecology and obstetrics	10 (62.5)	12 (75)	8 (50)	4 (25)	4 (25)
TCM pediatrics	11 (68.8)	13 (81.3)	5 (31.3)	7 (43.8)	7 (43.8)
TCM dermatology	14 (73.7)	17 (89.5)	14 (73.7)	12 (63.2)	12 (63.2)
TCM ENT^f^, ophthalmology	21 (56.8)	26 (70.3)	12 (32.4)	13 (35.1)	13 (35.1)
TCM traumatology	9 (45)	14 (70)	9 (45)	8 (40)	8 (40)
TCM acupuncture	56 (70)	60 (75)	43 (53.8)	34 (42.5)	33 (41.3)

a LOTS: lower-order thinking skills.

b HOTS: higher-order thinking skills.

c SAMC: single-answer multiple-choice.

d SAMRMC: single-answer, multiple-response multiple-choice.

e TCM: traditional Chinese medicine.

f ENT: ears, nose, and throat.

### Consistency Between AI-Generated Answers and Human-Curated Answers and Analysis of Incorrect Responses Provided by the GPT-4 Model

**Figure 5.**
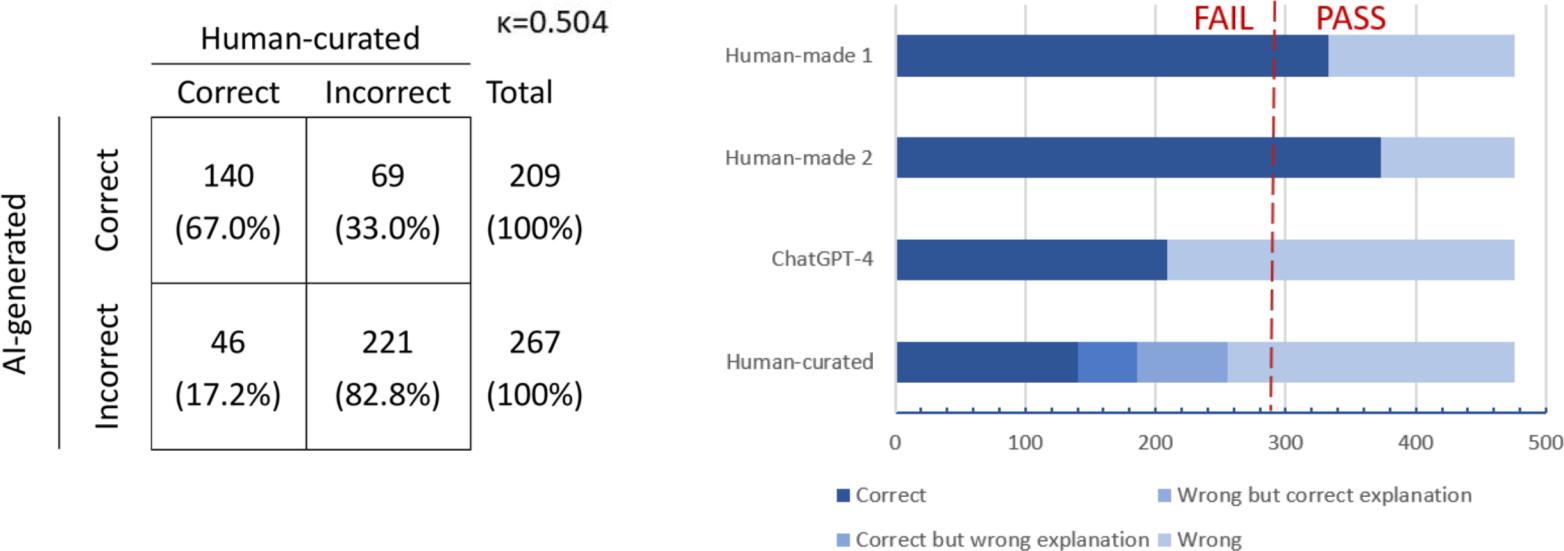
Accuracy rates of humans and ChatGPT-4 for TCM licensing examinations. The passing standard is an average score of 60. With 476 questions, the threshold is at least 286 correct answers (red dashed line). AI: artificial intelligence; TCM: traditional Chinese medicine.

The consistency between AI-generated and human-curated results was low (κ=0.504; Figure 5). After human review, the accuracy of the human-curated answers showed an overall trend of slight decrease, except for some minor increases in basic pharmacology and formulation, TCM pediatrics, and TCM otorhinolaryngology and ophthalmology. The accuracies for the remaining specialties were slightly lower, ranging from 43.9% to 40.3% (Table 2, Figures5  6). For human reviewer 1, discrepancies were observed between AI-generated responses and those reviewed by humans, with 23.96% (115 of 480 questions) of the answers provided by AI conflicting with its own explanations. For 33% of correctly answered questions (69 of 209 questions), the AI provided an incorrect explanation, indicating a scenario of “correct answer, incorrect explanation.” Conversely, for 17% of incorrectly answered questions (46 of 267 questions), the AI provided a correct explanation, suggesting a case of “incorrect answer, correct explanation.” This reduced the overall accuracy of the AI model to 43.9%.

**Figure 6.**
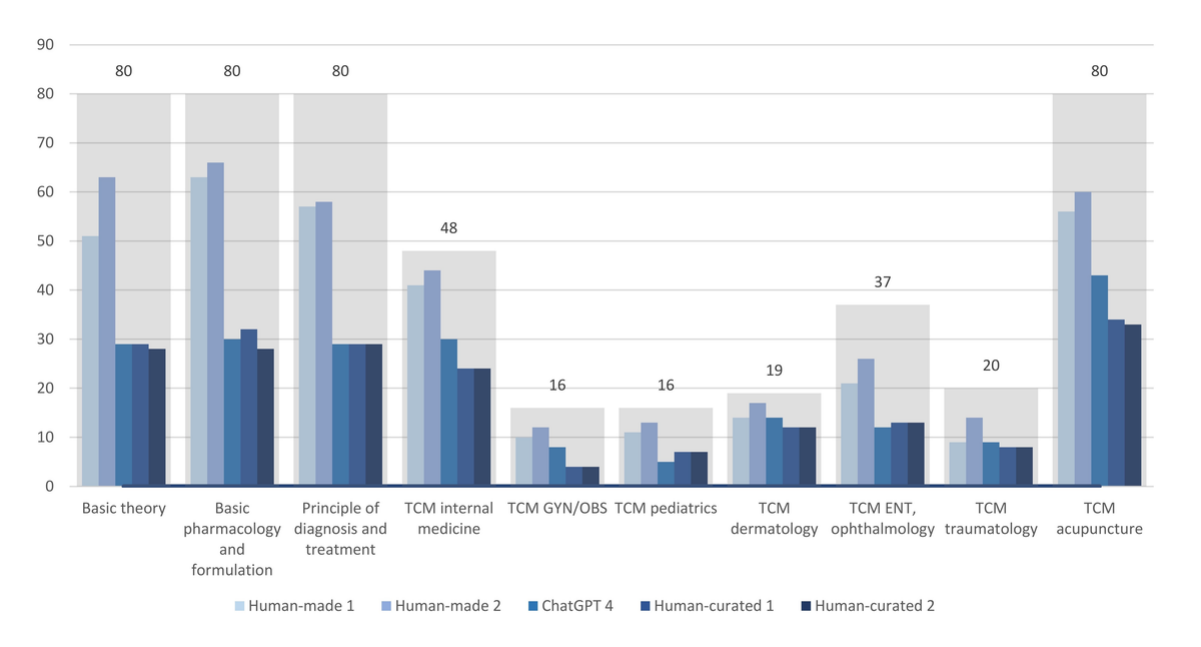
Performance of humans and ChatGPT-4 across various subjects. ENT: ears, nose, and throat; GYN/OBS: gynecology/obstetrics; TCM: traditional Chinese medicine.

**Figure 7.**
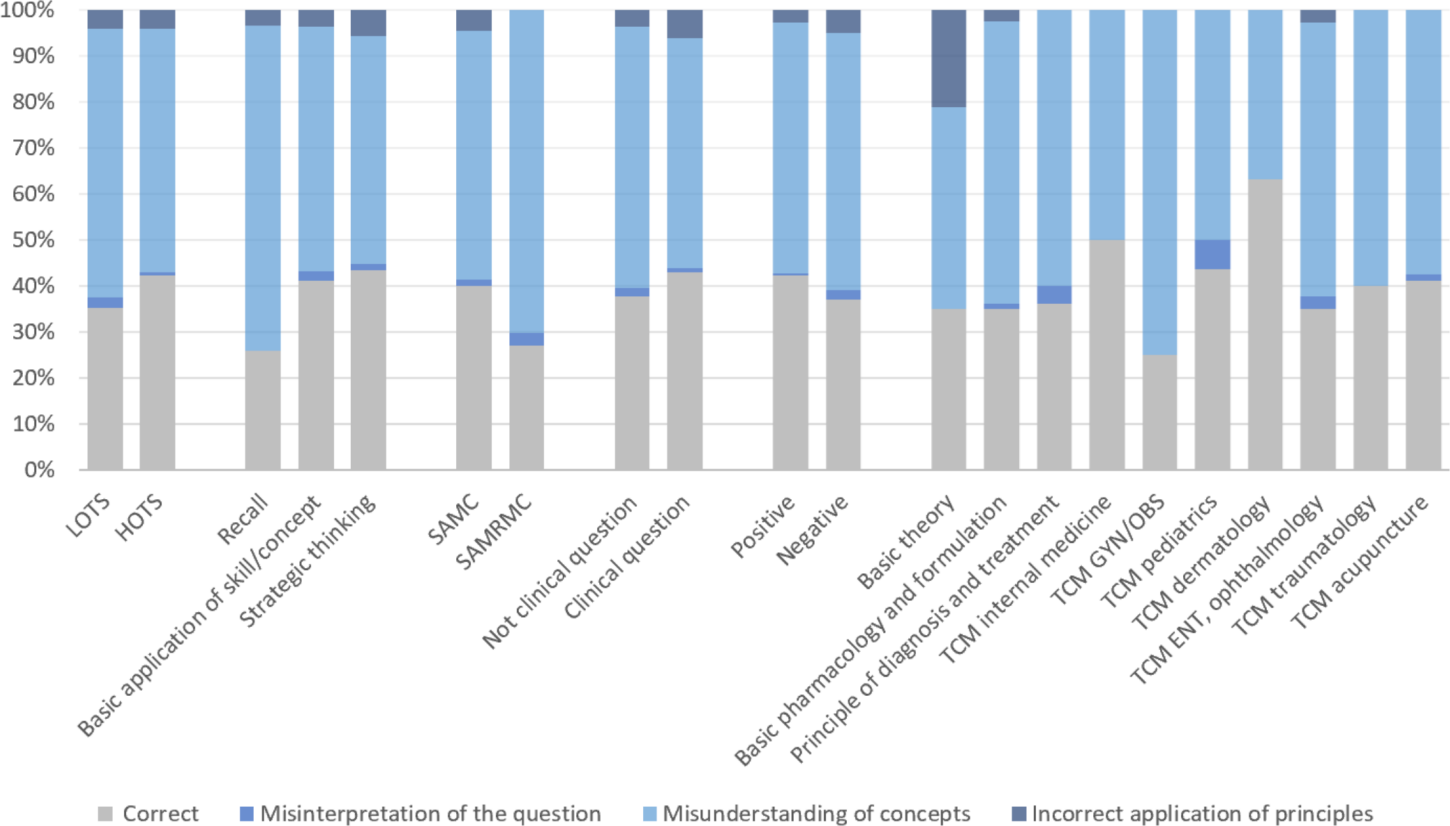
Distribution of reasons for incorrect answers provided by ChatGPT-4. ENT: ears, nose, and throat; GYN/OBS: gynecology/obstetrics; HOTS: higher-order thinking skills; LOTS: lower-order thinking skills; SAMC: single-answer multiple-choice; SAMRMC: single-answer, multiple-response multiple-choice; TCM: traditional Chinese medicine.

We further analyzed the reasons responsible for the incorrect answers provided by the GPT. For this purpose, we categorized the potential reasons for these errors into 3 types: misinterpretation of the question (failing to understand the question), misunderstanding of concepts (lacking knowledge of the topic), and incorrect application of principles (the content is correct, but it does not answer the question). The results revealed that most of the errors (263/476, 55.3%) were attributed to the misunderstanding of concepts (Table 4, Figure 7). However, a closer examination of the different characteristics of the questions indicated that misunderstanding of concepts was more common in LOTS, recall, and SAMRMC compared to their counterparts. The second most common cause of error was incorrect application of principles (20/476, 4.2%), followed by misinterpretation of questions (7/476, 1.5%).

**Table 4.** Reasons responsible for incorrect artificial intelligence–generated responses (human-curated).

	Correct (n=186)	Misinterpretation of the question (n=7)	Misunderstanding of concepts (n=263)	Incorrect application of principles (n=20)	*P* value
**Bloom’s cognitive level**					.25
LOTS^a^	75 (40.3)	5 (71.4)	124 (47.1)	9 (45)	
HOTS^b^	111 (59.7)	2 (28.6)	139 (52.9)	11 (55)	
**Depth of knowledge**					.06
Recall	22 (11.8)	0 (0)	60 (22.8)	3 (15)	
Basic application of skill/concept	102 (54.8)	5 (71.4)	132 (50.2)	9 (45)	
Strategic thinking	62 (33.3)	2 (28.6)	71 (27)	8 (40)	
**Type of questions**					.16
SAMC^c^	176 (94.6)	6 (85.7)	237 (90.1)	20 (100)	
SAMRMC^d^	10 (5.4)	1 (14.3)	26 (9.9)	0 (0)	
**Vignette style question**					.39
Without clinical vignette	137 (73.7)	6 (85.7)	206 (78.3)	13 (65)	
With clinical vignette	49 (26.3)	1 (14.3)	57 (21.7)	7 (35)	
**Polarity of question**					.28
Positive	76 (40.9)	1 (14.3)	98 (37.3)	5 (25)	
Negative	110 (59.1)	6 (85.7)	165 (62.7)	15 (75)	
**Subjects**					<.001
Basic theory	28 (15.1)	0 (0)	35 (13.3)	17 (85)	
Basic pharmacology and formulation	28 (15.1)	1 (14.3)	49 (18.6)	2 (10)	
Principle of diagnosis and treatment	29 (15.6)	3 (42.9)	48 (18.3)	0 (0)	
TCM^e^ internal medicine	24 (12.9)	0 (0)	24 (9.1)	0 (0)	
TCM gynecology and obstetrics	4 (2.2)	0 (0)	12 (4.6)	0 (0)	
TCM pediatrics	7 (3.8)	1 (14.3)	8 (3.0)	0 (0)	
TCM dermatology	12 (6.5)	0 (0)	7 (2.7)	0 (0)	
TCM ENT^f^, ophthalmology	13 (7)	1 (14.3)	22 (8.4)	1 (5)	
TCM traumatology	8 (4.3)	0 (0)	12 (4.6)	0 (0)	
TCM acupuncture	33 (17.7)	1 (14.3)	46 (17.5)	0 (0)	

a LOTS: lower-order thinking skills.

b HOTS: higher-order thinking skills.

c SAMC: single-answer multiple-choice.

d SAMRMC: single-answer, multiple-response multiple-choice.

e TCM: traditional Chinese medicine.

f ENT: ears, nose, and throat.

## Discussion

### Performance of ChatGPT in Medical Examinations

This is the first study to test the capabilities of ChatGPT in TCM examinations. ChatGPT has undergone rigorous testing for its proficiency in medical examinations. Nonetheless, its effectiveness in TCM licensing examinations remains unexplored. Hence, this study fills a research void by examining the capability of an advanced language model like ChatGPT in the context of TCM. Generally, most studies indicate ChatGPT can meet the medical examination pass standards. For example, ChatGPT 3.5 scored around the pass mark on the United States Medical Licensing Examination [14] and exhibited strong performance in specialties such as radiation oncology and neurosurgery [27,28]. GPT-4 surpassed 70% in its score for UK medical licensing examinations [12], and its competency extends to examinations in different languages. For example, GPT 3.5 typically scored around the passing mark on the Japanese nursing examinations [16] and Korean medical student parasitology examinations [29]. Although GPT-3.5 Turbo is not yet capable, GPT-4 passed the medical licensing examinations of China [30,31] and achieved 88.6% accuracy in the equivalent examinations of Saudi Arabia [32]. Interestingly, it even outperformed human residents in the residency training examinations of Japan [33].

Published research has identified 2 trends in this setting. First, GPT-4 surpasses GPT-3.5 in identical medical examinations, as demonstrated in medical student finals in Poland [34] and the medical licensing examinations of Peru [35]. A systematic review and meta-analysis of ChatGPT use in medical licensing examinations worldwide observed similar results [36]. Second, ChatGPT models showed higher accuracy when answering questions translated into English compared with the original language [34,37]. In Taiwan, traditional Chinese is the language used for medical licensing examinations. Despite this disadvantage, ChatGPT performed near the pass threshold for the nursing [38] and pharmacy licensing examinations in Taiwan [15]; translating pharmacy examination questions into English indeed improved scores across all subjects [15]. Thus, it was hypothesized that GPT-4 would perform similarly in TCM licensing examinations. However, the results were surprising. The study used the first 2022 TCM licensing examinations in Taiwan as a case study to assess the performance of the model. GPT-4 failed the exam with an overall accuracy of 43.9%; following human revision of AI-provided explanations, the accuracy further decreased to 40.3% (human 1) and 39.1% (human 2). These results underscore the need for further research and development on the application of AI models to TCM examination preparation and highlight the existing knowledge gap. The reasons behind these outcomes merit further investigation.

### Challenges Encountered by ChatGPT When Answering Medical Questions

Previous literature has discussed the shortcomings and challenges of ChatGPT in answering examination questions, including a decreased proficiency in languages other than English [34,37], AI “hallucinations” originating from erroneous data [10,38], and proficiency limited to certain types of questions [13,39]. The tendency for ChatGPT to be less proficient in answering questions posed in languages other than English stems from the fact that ChatGPT is an LLM trained primarily on English language data, which includes a wide variety of sources such as books, websites, and news articles [6]. The questions for TCM licensing examinations are not presented in English. Although ChatGPT can fluently interact in traditional Chinese, its responses to medical examination questions, which require specific expertise and have standard answers, may reveal its inadequacies. AI “hallucinations” indicate a tendency to produce “hallucinations” or factually incorrect content due to incorrect data. This poses the risk of generating misleading or fabricated information, which complicates the use of AI as a reliable self-learning tool [7,10]. We also encountered seemingly plausible but incorrect content in AI-generated responses in our research. We even found that verifying the authenticity of these answers is more time-consuming and requires deeper professional knowledge than the questions themselves. Our study also showed that ChatGPT had higher, albeit not statistically significant, accuracy rates for questions posed such as SAMC (n=197, 44.9%) and presented with clinical vignettes (n=52, 45.6%). This trend aligns with findings of previous studies, such as a lower proficiency in multiple-choice questions [13] and a poorer aptitude for conceptual questions compared with clinical scenarios [39]. Despite these limitations, which we have also encountered, other research has shown that ChatGPT can pass examinations. Therefore, the use of ChatGPT in the context of TCM may pose its own unique set of challenges and necessitates further investigation.

### Challenges Encountered by ChatGPT When Answering TCM Examination Questions

We identified 3 main reasons for incorrect answers according to AI-generated responses, namely misinterpretation of the question, misunderstanding of concepts, and incorrect application of principles. Misunderstanding of concepts was the most prevalent, especially in questions with lower cognitive demand such as recall and LOTS, as well as in questions where a single item encompasses multiple questions (eg, SAMRMC), indicating either a lack of knowledge or incorrect knowledge. We believe that this primarily stems from 2 factors. First, the database for TCM is currently incomplete. Second, compared with Western medicine, TCM is often considered alternative medicine. If an LLM such as ChatGPT answers questions based solely on the Western medical knowledge system, then TCM content may be ignored. Additionally, TCM focuses on personalized treatment without a golden standard, leading to the absence of definitive answers for the same disease.

The incomplete TCM database is due to challenges such as insufficient data, lack of standardization, and unrepresentative data sources. Although the specific TCM data that ChatGPT uses for training are unclear, it is evident that the current online data for TCM are significantly less comprehensive than those for Western medicine. For instance, a bibliometric analysis over the past 20 years did not show a significant presence of TCM-related keywords in the context of pediatric allergic rhinitis [40]. However, the usage rate of TCM for allergic diseases in Taiwan is approximately 30%‐50% [41]. Therefore, a model constructed based on such a database is likely to exhibit discrepancies with reality. Furthermore, online data often contain inaccuracies or incomplete information. Previous research has shown that uncleaned training texts can affect performance and could underpin the subpar performance of the trained model [42].

It is important to note that, due to challenges in translation and cultural appropriation, certain medical terms have different connotations in the TCM and Western medical systems. However, ChatGPT tends to interpret these terms with a preference for their meanings within Western medicine. For instance, in some AI-generated responses, the TCM term for “肝” was mistakenly translated and described as the physical organ “liver” in Western medicine. Similarly, the term for “瘧” in TCM was translated and described as “malaria” in some AI-generated responses. The understanding of “肝” in TCM is not entirely the same as in modern medicine, and “瘧” in TCM refers to a broad category of symptoms similar to malaria but not restricted to infections caused by *Plasmodium*.

The crux of TCM is personalized treatment, which is antithetical to gold-standard treatments. Hence, multiple therapeutic approaches may exist for the same disease. If the examination questions do not specify a particular scope or clear criteria, there may be no standard answer or multiple possible solutions. This study revealed that the decrease in the overall accuracy rate after human review was primarily driven by a reduction in accuracy for LOTS questions, whereas the accuracy rate for HOTS remained stable or even increased. Regarding DOK, the decrease in accuracy following human review was primarily in recall, with less of a decrease noted in more advanced DOK (eg, basic application of skill/concept, strategic thinking). This suggests that GPT-4 is more adept at providing detailed explanations for complex logical reasoning questions, as opposed to simple memorization, which might be influenced by incorrect information. In addition, if users intend to use GPT to answer TCM questions, they should be particularly cautious of potential hallucinations in lower cognitive demand questions.

Our study revealed that the GPT-4 model is currently unable to pass the TCM licensing examinations. This research underscores the limitations of the performance of AI in TCM licensing examinations, as well as illuminates broader challenges within the realm of integrating TCM knowledge into AI development.

### Limitations

Although this study provides valuable insights into the use of the GPT-4 model for TCM licensing examination preparation, several limitations have been identified. The focus solely on the GPT-4 model of ChatGPT might neglect the complexities and potential capabilities of other recently developed AI-driven language models, such as Claude 3 by Anthropic, Bard (Gemini Pro) by Google, or LLaMa2 by Facebook. Notably, we did not use expert-level AI, such as Med-PaLM by Google [43]. Moreover, we did not use other traditional Chinese-language LLMs, such as Taiwan-LLM [44,45]. Nevertheless, GPT models are the most widely used and studied models, and it is necessary to use the same tool to facilitate comparisons with other research studies [36].

Considering the cultural context specific to the TCM licensing examination of Taiwan, the generalizability of our findings to different regions or educational systems may be limited. Notably, model performance may change over time, indicating that our results may not be replicated in the future. This study also did not account for potential inconsistencies in responses provided by ChatGPT to identical queries during different sessions. However, this issue could be minimized by explicitly setting the parameters of ChatGPT.

Additionally, the difficulty of each exam can vary, which might affect ChatGPT’s performance. However, the difficulty is generally controlled and, as a national exam, the pass rates have been stable over the years [46]. Previous exam questions could potentially be part of the GPT model’s training data (with a knowledge cutoff date of September 2021), introducing bias. Therefore, we only used the first exam of 2022 to mitigate this issue.

### Implications for Practice and Future Research

This study investigated the use of the GPT-4 model for TCM licensing examination preparation. The findings revealed that AI-driven tools are not yet valuable assets for TCM educators and students. The observed limitations (ie, often providing responses based on incorrect facts) highlight the need for further development before this model can be effectively used as a self-learning tool. As the AI field continues to advance with the introduction of new models, educators must stay informed and utilize the most effective tools while being cognizant of their limitations. This study sets the stage for 2 potential research directions. In terms of TCM, considering the suboptimal examination results, we speculate that the primary drawback lies in the quality of the front-end data. Future improvements may include incorporating ancient TCM texts and customizing training for LLMs.

We must deliberately incorporate relevant resources into our training database materials, such as textbooks on TCM in Chinese and ancient TCM texts. Currently, the majority of descriptions and knowledge regarding TCM are in Chinese. When these data are published in journals or translated into English, they often adopt the framework and language of modern medicine as a medium for knowledge transmission. This approach tends to underemphasize the original content of TCM, which is mostly documented in Chinese literature. Therefore, the inclusion of TCM materials in LLM training and the standardization of TCM should be targeted for improvement.

Tailoring training data for LLMs presents another promising avenue for improvement. TCM comprises different schools, suggesting that narrowing the knowledge domain could be more advantageous. Hence, to excel in TCM, developing specialized ChatGPT models or custom LLMs might be a beneficial strategy. Considering the current limitations in enhancing the database, integrating specific prompts offers an alternative solution. For example, the chain-of-thoughts method, used in LLMs for complex problem-solving, articulates intermediate steps in reasoning. This approach is particularly effective for models with extensive parameters, enhancing their ability to manage multistep tasks [26]. It has been confirmed that this method can also improve the performance of ChatGPT in medical examinations [47]. Hence, the adoption of chain-of-thoughts may be a viable strategy to address the complexity of TCM examinations. Additionally, previous research indicated that restricting ChatGPT to a single response in a Basic Life Support examination may introduce bias. When ChatGPT generates 3 responses per question, it successfully passes the examination. Moreover, rephrasing incorrectly answered questions as open-ended questions significantly boosts the accuracy of ChatGPT. This implies that open-ended questioning or multiple inquiries might be more effective than single-choice formats [48].

### Conclusion

Our study represents the first comprehensive assessment of the performance of ChatGPT in TCM licensing examinations. Despite advances in AI and its success in various medical licensing tests, ChatGPT demonstrated a limited ability to accurately respond to TCM examination questions, achieving an overall accuracy rate significantly lower than that of its human counterparts. This shortfall underscores the challenges posed by the unique concepts and terminologies of TCM, highlighting a significant knowledge gap in the understanding of TCM principles by AI. Our findings call for further advancements in AI training, specifically tailored toward the intricate domain of TCM, to enhance its utility in this specialized field of medicine.

## Supplementary material

10.2196/58897 Multimedia Appendix 1 List of the 5 factors, data definitions, and source citations.

10.2196/58897 Multimedia Appendix 2 Examples of single-answer multiple-choice and single-answer, multiple-response multiple-choice questions.

10.2196/58897 Multimedia Appendix 3 Examples of the prompt used to generate responses from questions.

10.2196/58897 Multimedia Appendix 4 Examples of the prompts used to generate responses from questions with explanations for each item.

## Abbreviations

AI: artificial intelligence
aOR: adjusted odds ratio
DOK: depth of knowledge
ENT: ears, nose, and throat
GPT: generative pretrained transformer
GYN/OBS: gynecology/obstetrics
HOTS: higher-order thinking skills
LLM: large language model
LOTS: lower-order thinking skills
SAMC: single-answer multiple-choice
SAMRMC: single-answer, multiple-response multiple-choice
TCM: traditional Chinese medicine
