# Supplementary material for: Performance of ChatGPT-4 on Taiwanese Traditional Chinese Medicine Licensing Examinations: Cross-Sectional Study
Source: JMIR Med Educ. 2025 Mar 19;11:e58897. doi: 10.2196/58897 (PMC11939018; doi:10.2196/58897)
Supplement: Multimedia Appendix 1 [file mededu-v11-e58897-s001.docx]

**Supplementary Table S1.** List of the five factors, data definitions, and source citations.

| Factors | Data definitions | Source citation |
| --- | --- | --- |
| Cognitive level | **Lower-order thinking skills (LOTS) :**  **include remembering, understanding, and applying knowledge to questions**  Remember - Retrieving relevant knowledge from long-term memory  Understand - Determining the meaning of instructional messages, including oral, written, and graphic communication  Apply - Carrying out or using a procedure in a given situation. | D. R. Krathwohl, "A revision of Bloom's taxonomy: An overview," Theory into practice, vol. 41, no. 4, pp. 212-218, 2002. |
|  | **Higher-order thinking skills (HOTS):**  **include further analyzing, evaluating, and creating after learning**  Analyze - Breaking material into its constituent parts and detecting how the parts relate to one another and to an overall structure or purpose.  Evaluate - Making judgments based on criteria and standards.  Create - Putting elements together to form a novel, coherent whole or make an original product. |  |
| Depth of knowledge (DOK) | **Recall**:  recall of information | N. L. Webb, "Depth-of-knowledge levels for four content areas," Language Arts, vol. 28, no. March, pp. 1-9, 2002. |
|  | **Concept**: the engagement of some mental processing beyond recalling or reproducing a response |  |
|  | **Strategic thinking**:  reasoning, planning, using evidence, and a higher level of thinking than the previous two levels |  |
|  | **Extended thinking**:  complex reasoning, experimental design, and planning likely require extended time for scientific investigation or completing multiple assessment steps |  |
| Type of questions | **Single-answer multiple-choice (SAMC)**:  questions had only one most appropriate answer |  |
|  | **Single-answer multiple-response multiple-choice (SAMRMC)**:  questions require the tester to choose the most appropriate answer composed of multiple correct options provided in each question |  |
| Vignette style | **Without clinical vignette**:  content of a question presents clinical scenarios |  |
|  | **With clinical vignette**:  content of a question don’t present clinical scenarios |  |
| Polarity of questions | **Positive** :  solicits the correct or affirmative answer |  |
|  | **Negative** :  demands the identification of the incorrect or negative answer |  |
