# Supplementary material for: Performance of ChatGPT-4 on Taiwanese Traditional Chinese Medicine Licensing Examinations: Cross-Sectional Study
Source: JMIR Med Educ. 2025 Mar 19;11:e58897. doi: 10.2196/58897 (PMC11939018; doi:10.2196/58897)
Supplement: Multimedia Appendix 2 [file mededu-v11-e58897-s002.docx]

**Supplementary Table S2.** Examples of single-answer multiple-choice (SAMC) and single-answer multiple-response multiple-choice (SAMRMC).

| Question type | Content | Answer |
| --- | --- | --- |
| SAMC | 一個人出現說夢話、夢遊，甚至出現幻覺、幻聽等症狀，依《難經》五臟藏七神之理論，此問題可歸因下列何臟腑？  A.肝 B.脾 C.肺 D.腎  Translation:  A person exhibits symptoms such as sleep talking, sleepwalking, and even hallucinations and auditory hallucinations. According to the theory of the five viscera and seven spirits in the ‘Nan Jing’, which of the following organs can this problem be attributed to?  A. Liver B. Spleen C. Lung D. Kidney | A |
| SAMRMC | 人體之氣的主要來源包括下列何者？①先天之精氣 ②臟腑之氣 ③自然界之清氣 ④水穀之精氣⑤宗氣 A.②③④ B.①②⑤ C.①③④ D.②③⑤  Translation:  What are the main sources of qi in the human body? ①Prenatal essence qi ②Zang-fu qi ③Natural clear qi ④Water-grain essence qi ⑤Ancestral qi  A.②③④ B.①②⑤ C.①③④ D.②③⑤ | C |
