# Supplementary material for: Performance of ChatGPT-4 on Taiwanese Traditional Chinese Medicine Licensing Examinations: Cross-Sectional Study
Source: JMIR Med Educ. 2025 Mar 19;11:e58897. doi: 10.2196/58897 (PMC11939018; doi:10.2196/58897)
Supplement: Multimedia Appendix 3 [file mededu-v11-e58897-s003.docx]

**Supplementary Table S3.** Examples of the prompt used to generate responses from questions.

| Example | Content | Answer from GPT-4 |
| --- | --- | --- |
| SAMC | 下列何種藥物，中醫用於熱結便秘，其主成分也用於現行西醫臨床上的便秘？  A.番瀉葉 B.何首烏 C.甘遂 D.巴豆  Translation:  Which of the following drugs is used in traditional Chinese medicine for heat-induced constipation and has its main ingredient also used in current Western medicine for constipation? A. Senna leaf B. He Shou Wu C. Gan Sui D. Croton seed  **Prompt: think step-by-step but show me only the answer, do not explain it**  GPT-4  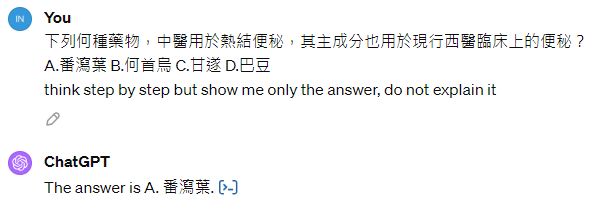 | A |
| SAMRMC | 關於小兒暑邪感冒的症狀，下列何者最適當？①發熱重，多汗 ②身重困倦，胸悶泛惡 ③食欲不振，或嘔吐、腹瀉 ④舌苔薄白或膩、質淡紅、脈浮數  A.①② B.②③ C.②④ D.①④  Translation:  Regarding the symptoms of summer-heat cold in children, which of the following is most appropriate? ① High fever, heavy sweating ② Feeling heavy and fatigued, chest tightness, and nausea ③ Loss of appetite, or vomiting and diarrhea ④ Thin white or greasy tongue coating, light-red tongue body, floating and rapid pulse  A.①② B.②③ C.②④ D.①④  **Prompt: think step-by-step but show me only the answer, do not explain it**  GPT-4  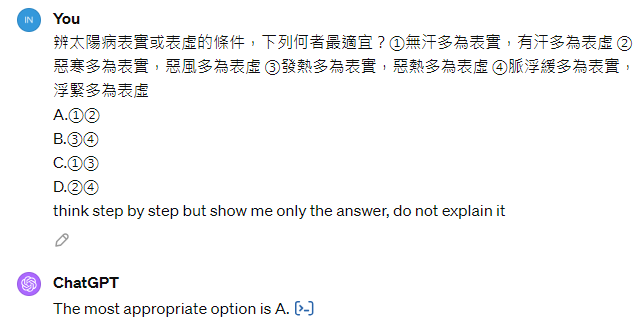 | A |

Abbreviations: GPT: Generative Pre-trained Transformer; SAMC: single-answer multiple-choice; SAMRMC: single-answer multiple-response multiple-choice.
