# Supplementary material for: Performance of ChatGPT-4 on Taiwanese Traditional Chinese Medicine Licensing Examinations: Cross-Sectional Study
Source: JMIR Med Educ. 2025 Mar 19;11:e58897. doi: 10.2196/58897 (PMC11939018; doi:10.2196/58897)
Supplement: Multimedia Appendix 4 [file mededu-v11-e58897-s004.docx]

**Supplementary Table S4.** Examples of the prompts used to generate responses from questions with explanations for each item.

| Example | Content | Explanations and answers from GPT-4 |
| --- | --- | --- |
| SAMC | 下列何者不是牙宣病的常見主因？  A.心脾積熱  B.胃火上蒸  C.腎陰虛損  D.氣血不足  Translation:  Which of the following is not a common primary cause of gum disease?  A. Accumulation of heat in the heart and spleen  B. Stomach fire steaming upwards  C. Damage due to deficiency of kidney yin  D. Insufficiency of Qi and blood  **Prompt: explain each item** | 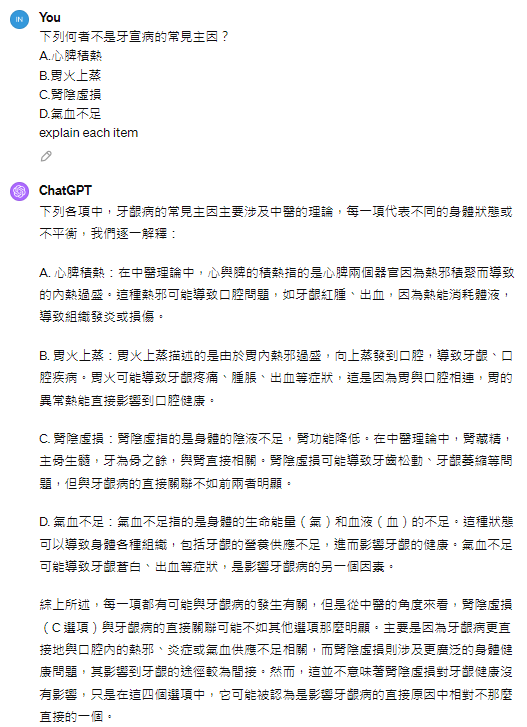  GPT’s answer is C |
| SAMRMC | 關於小兒暑邪感冒的症狀，下列何者最適當？①發熱重，多汗 ②身重困倦，胸悶泛惡 ③食欲不振，或嘔吐、腹瀉 ④舌苔薄白或膩、質淡紅、脈浮數  A.①② B.②③ C.②④ D.①④  Translation:  Which of the following is the most appropriate symptom of heat evil cold in children? ① Severe fever, excessive sweating ② Heaviness and fatigue, chest tightness and nausea ③ Loss of appetite, or vomiting, diarrhea ④ Thin white or greasy tongue coating, light-red tongue body, floating and rapid pulse  A. ①② B. ②③ C. ②④ D. ①④  **Prompt: explain each item** | 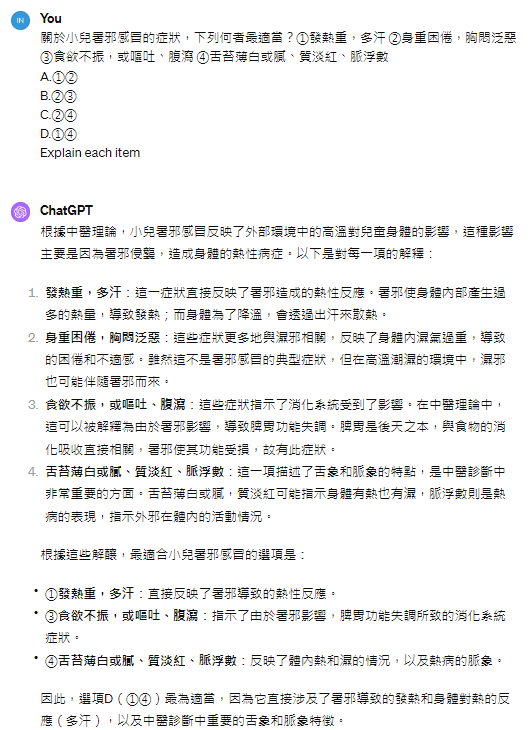  GPT’s answer is D |

Abbreviations: GPT: Generative Pre-trained Transformer; SAMC: single-answer multiple-choice; SAMRMC: single-answer multiple-response multiple-choice.
